# Development of a Novel Enzyme-Linked Immunosorbent Assay Targeting a Neo-Epitope Generated by Cathepsin-Mediated Turnover of Type III Collagen and Its Application in Chronic Obstructive Pulmonary Disease

**DOI:** 10.1371/journal.pone.0170023

**Published:** 2017-01-11

**Authors:** Daniel Guldager Kring Rasmussen, Jannie Marie Bülow Sand, Morten Asser Karsdal, Federica Genovese

**Affiliations:** 1 Nordic Bioscience, Biomarkers and Research, Herlev, Denmark; 2 University of Southern Denmark, Institute of Molecular Medicine, Cardiovascular and Renal Research, Institute of Clinical Research, Odense, Denmark; Stanford University, UNITED STATES

## Abstract

A high level of extracellular matrix (ECM) turnover characterizes several lung diseases with fibrotic features. Type III collagen is one of the most abundant collagens in lung parenchyma, and cathepsins play a role in lung pathology, being responsible for tissue remodeling. In this study, we explore the diagnostic features of neo-epitope fragments of type III collagen generated by cathepsins that could reflect the pathological tissue turnover in patients with different diseases. A novel enzyme-linked immunosorbent assay (ELISA) measuring cathepsins B, L, S and K -generated type III collagen fragments (C3C) was developed for assessment in serum and plasma. The assay was biologically validated in serum from patients with chronic obstructive pulmonary disease (COPD). Serological levels of C3C were significantly elevated in patients with COPD compared to healthy controls (p = 0.0006). Levels of C3C in serum and heparin plasma of COPD patients had a highly significant correlation (R^2^ = 0.86, p<0.0001). The data suggests that the C3C fragment is elevated in patients with COPD compared to healthy controls.

## Background

Fibrillar collagens, such as type I and III collagen, are some of the most prominent collagens in the extracellular matrix (ECM) of the lung [1–3]. Fibrillar collagens provide tensile strength which enables enlargement of the lung components such as the alveoli, vessels, and connective tissue sheaths. An important requirement for balanced remodeling of the ECM is a tight control of enzymes involved in its turnover. Fibrotic diseases have an imbalance between formation and degradation which leads to an altered composition of the ECM thereby causing an abnormal tissue function [4,5]. Plenty of studies have presented findings suggesting the involvement of cathepsins in tissue turnover in various organs, such as the lungs. As an example for pathologies of the lung, cathepsins are upregulated in patients with chronic obstructive pulmonary disease (COPD)[6,7], idiopathic pulmonary fibrosis (IPF) [8] and lung cancer [9–11]. Some studies suggested that cathepsins B and K are implicated in the mechanisms leading to invasion of lung adenocarcinomas [10,12]. In patients with COPD, cathepsin S and L were shown to be involved in degradation of the extracellular matrix mediated by macrophages [13,14]. Structural changes resembling COPD were seen in two transgenic mouse strains overexpressing either IL-13 or IFN- γ [15,16]. In these models cathepsins B, L, S, and K were upregulated and caused lung epithelial apoptosis, lung inflammation, and airspace enlargement [15,16]. In an attempt to assess dynamic turnover of the extracellular matrix by cathepsins, we developed a novel neo-epitope assay, which utilizes a monoclonal antibody targeting a specific neo-epitope of type III collagen generated by cathepsins B, L, S and K and measured the levels of this fragment in samples from patients with different lung pathologies.

## Methods

### Reagents

All reagents used in the experiments were high standard chemicals from Merck (Whitehouse Station, NJ, USA) and Sigma Aldrich (St. Louis, MO, USA). The synthetic peptides used for immunization and assay development were 1) Immunogenic peptide: GLPGTGGPPG-GGC-KLH, 2) Screening peptide: GLPGTGGPPGK-Biotin, 3) Standard peptide: GLPGTGGPPG, 4) Elongated peptide: QGLPGTGGPPG, and 5) Nonsense peptide: KNGETGPQGP. All synthetic peptides were purchased from the Chinese Peptide Company, Beijing, China.

### Monoclonal antibody development

**Fig 1:**
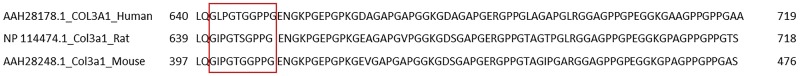
Sequence alignment between the alpha-1 chain of type III collagen in humans, rats and mice. The antibody recognizes the neo-epitope starting from residue 642 to 651 in the human protein (ie, GLPGTGGPPG). 90% homology was observed for the human and mouse sequence due to an amino acid change at the second residue in the target sequence (L → I). 80% sequence homology was observed between the human and rat sequence due to two amino acid changes at the second (L → I) and sixth (G → S) position of the target sequence.

The amino acid sequence 642’-GLPGTGGPPG-’651 located in the α1 chain of type III collagen was found in a proteomic analysis of patients with coronary artery disease [17]. It is 90% homologous between human and mouse and 80% homologous between human and rat (Fig 1). Generation of monoclonal antibodies was carried out as described previously [18]. Immunization was initiated by subcutaneous injection of 200 μl emulsified antigen and 50 μg immunogenic peptide (ie, GLPGTGGPPG-GGC-KLH) in 4–5 week old Balb/C mice using Freund’s incomplete adjuvant. The immunizations were repeated every 2 weeks until stable serum titer levels were reached. The mouse with the highest serum titer was selected for fusion. The mouse rested for a month and was then boosted intravenously with 50 μg immunogenic peptide in 100 μl 0.9% NaCl solution three days before isolation of the spleen. The spleen cells were fused with SP2/0 myeloma cells to produce hybridoma as described by Gefter and co-workers [19] and cloned in culture dishes using the semi-solid medium method. The clones were plated into 96-well microtiter plates for further growth employing the limited dilution method to secure monoclonal growth. The supernatants were screened for reactivity against standard peptide and native material in an indirect ELISA using streptavidin-coated plates. GLPGTGGPPG-K-Biotin was used as screening peptide, while the standard peptide GLPGTGGPPG was used as a calibrator to test for further specificity of clones.

### Clone characterization

The clone was characterized as previously described by Nielsen *et al* [18]. Native reactivity and affinity for the standard peptide were assessed using different biological materials such as human serum, heparin plasma and EDTA plasma purchased from a commercial supplier (Valley Biomedical, VA 22602, USA). Antibody specificity was tested in a preliminary assay using a nonsense and elongated peptides (ie, KNGETGPQGP and QGLPGTGGPPG, respectively). The isotype of the monoclonal antibody was determined using the Clonotyping System-HRP kit, cat. 5300–05 (Southern Biotech, Birmingham, AL, USA).

### C3C ELISA

Supernatant from antibody-producing hybridoma was collected and the monoclonal antibody was purified using HiTrap affinity columns (GE Healthcare Life Science, Little Chalfront, Buckinghamshire, UK) and labeled with horseradish peroxidase (HRP) using Lightning-Link^™^ HRP Conjugation Kit (Innova Biosciences, Babraham, Cambridge, UK), according to the manufacturer’s instructions.

The C3C competitive ELISA procedure was as follows; streptavidin-coated plates (Roche, cat. 11940279) were incubated with 100 μl biotinylated-peptide for 30 min at 20°C with shaking. Plates were washed five times in washing buffer (20 mM TRIS, 50 mM NaCl, pH 7.2). Sample/standard/control (20 μl) was added and followed immediately by addition of 100 μl HRP labeled monoclonal antibody and incubated for 1h at 20°C with shaking. After incubation, plates were washed five times in washing buffer. A volume of 100 μl 3,3’,5,5’-Tetramethylbenzidine (TMB, Kem-En-Tec Diagnostics) was added and incubated for 15 min at 20°C in the dark. To stop the enzyme reaction of TMB, 100 μl 0.1% sulphuric acid was added and the plate was analyzed in the ELISA reader at 450 nm with 650 nm as the reference (Molecular Devices, SpectraMax M, CA, USA). A standard curve was plotted using a 4-parametric mathematical fit model. Each ELISA plate included kit controls to monitor inter-assay variation. All samples were measured within the range of the assay. All samples below the level of lower limit of quantification (LLOQ) were assigned the value of LLOQ.

### Technical evaluation

The technical evaluation was performed as previously described by Nielsen *et al* [18]. Healthy human serum (n = 4), heparin plasma (n = 4), and EDTA plasma (n = 4) samples were used to determine linearity of dilution. The linearity was assessed by the percentage recovery of the undiluted sample. Antibody specificity was calculated as percentage of signal inhibition of 2-fold diluted standard peptide (GLPGTGGPPG), elongated peptide (QGLPGTGGPPG), and non-sense peptide (KNGETGPQGP). Lower limit of detection (LLOD) was calculated as the mean + 3*Standard Deviation (SD) of 21 determinations of standard K (i.e., buffer). Upper limit of detection (ULOD) was determined as the mean– 3*SD of 10 measurements of Standard A run in double determination. Lower limit of quantification (LLOQ) was determined as the lowest concentration measured in human serum with an error lower than 30%. The intra- and inter-assay variation was determined by 10 independent measurements of 7 quality control samples run in double determination. Accuracy of the assay was measured in healthy human serum samples spiked with standard peptide or a serum sample with a known high C3C concentration, and calculated as the percentage recovery of spiked peptide or serum in buffer. Interference was measured in healthy human serum spiked with hemoglobin (low = 0.155 mM, high = 0.310 mM), lipids (low = 4.83 mM, high = 10.98 mM), and biotin (low = 30 ng/ml, high = 90 ng/ml) and calculated as the percentage recovery of analyte in non-spiked serum.

### Analyte stability

The analyte stability was determined for three healthy human serum samples subjected to up to four freeze and thaw cycles. Analyte stability in the samples was calculated as the percentage recovery of sample undergone only one freeze/thaw cycle. Analyte stability was furthermore determined by incubation of three healthy human serum samples at either 4 or 20°C for 2, 4 and 24 hours and calculated as the percentage of the sample kept at -20°C (0 hour sample).

### Cleavage analysis

To assess which proteases were capable of generating the C3C neo-epitope, type III collagen was incubated for 24h at 37°C with selected matrix metalloproteases (MMP) 1, 3, 9, 10, 12, 13, 14 or 16, a disintegrin and metalloproteinase 10 (ADAM-10), ADAM with thrombospondin motifs (ADAM-TS) 1, 4 or 8, and cathepsins B, L, S or K (Table 1). Cleavage solutions contained final concentrations of 100 μg/mL type III collagen and 1 μg/mL protease. For activation of MMP-1, 3, 9, and -10, the proteases were pre-incubated with 1 mg/mL APMA in DMSO for 2 hours at 37°C. MMP-16 was activated by pre-incubation with furin, according to the manufacturer’s instructions. All MMP cleavage analysis were carried out in MMP buffer (150 mM NaCl, 50 mM Tris-HCl, 10 mM CaCl_2_, 10 μM ZnCl_2_, 0.05% (w/v) Brij-35, pH 7.5). ADAM-10 cleavage analysis was carried out in ADAM buffer (25 mM Tris, 2 μM ZnCl2, 0.005% (w/v) Brij-35, pH 9.0). ADAMTS-1, 4, and -8 cleavage analysis was carried out in ADAMTS buffer (50 mM Tris-HCL, 100 mM NaCl, 10 mM CaCl_2_, pH 7,5). Cathepsin K was activated by pre-incubation in activation buffer (100 mM NaAcetate, 10 mM DTT, 5 mM EDTA, pH 3.9) for 40 minutes at room temperature. All cleavage analysis utilizing cathepsins were performed in cathepsin buffer (50 mM MES, 2.5 mM EDTA, 5 mM DTT, pH 5.5). The cleavage reactions for MMPs were stopped by adding 5 mM EDTA in the ratio 1:100 (EDTA:cleavage solution). All solutions were immediately frozen until analysis.

**Table 1:** Products used for cleavage analysis.

Protein	Company (Distributor)	Cat. No.	Activation required
**Type III collagen**	Cell Sciences (Cell Sciences)	CRC160B	-
**MMP-1**	Calbiochem (VWR)	444208–5	Yes
**MMP-3**	Calbiochem (VWR)	PF063-10	Yes
**MMP-9**	Calbiochem (VWR)	444231–5	Yes
**MMP-10**	Enzo Life Sciences (SMS gruppen)	SE-329-0010	No
**MMP-12**	Abcam (Abcam)	ab54058	No
**MMP-13**	Calbiochem (VWR)	444287–5	No
**MMP-14**	Abcam (Abcam)	ab54060	No
**MMP-16**	R&D Systems (R&D Systems)	1785-MP	Yes
**ADAM-10**	Calbiochem (Merck)	PF124-20	No
**ADAMTS-1**	Abnova (Tebu-Bio)	H00009510-Q01	No
**ADAMTS-4**	Chemocon Int. (Merck Millipore)	CC1028	No
**ADAMTS-8**	Abnova (Tebu-Bio)	H00011095-Q01	No
**Cathepsin B**	Calbiochem (VWR)	219362–50	No
**Cathepsin L**	Calbiochem (VWR)	219402–25	No
**Cathepsin S**	Calbiochem (VWR)	219344–25	No
**Cathepsin K**	Calbiochem (VWR)	342001–10	Yes
**rhFurin**	R&D Systems (R&D Systems)	1503-SE	No

### Ethics statement

The work performed in mice was approved by the National Authority (The Animal Experiments Inspectorate) under approval number 2013-15-2934-00956. All animals were treated according to the guidelines for animal welfare.

A previously described cohort of COPD patients was used to assess the levels of C3C in serum and plasma of patients with COPD [20]. Inclusion criteria were a diagnosis of COPD made by a senior physician and FEV_1_ < 80% of predicted value. Exclusion criterion was an acute exacerbation of COPD leading to hospitalization within the previous four weeks. Sixty-eight subjects were included in the study. The study complies with the Declaration of Helsinki and Good Clinical Practice Guidelines, and has been approved by the local ethics committee (protocol number H-6-2013-014). All participants provided written informed consent before the performance of all study-related assessments. Levels of C3C in the COPD patients were compared to levels in commercially available control sera from healthy donors (Valley BioMedical, Winchester, VA, USA). Demographics of the cohort are shown in Table 2. Samples were all collected, processed, and stored in a similar fashion until analyses. All measurements were performed blinded.

**Table 2:** Patient demographics.

	COPD (n = 68)	Healthy control (n = 20)	p-value
**Age**	71.1 (9.0)	41.2 (12.2)	<0.0001
**Male, n (%)**	40 (59)	14 (70)	0.52
**BMI**	24.5 (6.1)	NA	-
**FEV**_***1***_**% of predicted value**	40.4 (16.3)	NA	-
**FEV**_***1***_**/FVC ratio%**	49.6 (0.14)	NA	-

Data is presented as mean (SD) unless otherwise stated. Comparison of age in healthy controls versus COPD patients was performed using the Mann-Whitney unpaired t-test. P-values below 0.05 were considered significant. The difference in gender between the groups was assessed by a Chi-squared test.

**Abbreviations**: BMI; body mass index; COPD, chronic obstructive pulmonary disease; FEV1, forced expiratory volume in one second; FVC, forced vital capacity.

### Statistical analyses

Comparison of age in healthy controls versus COPD patients was performed using the Mann-Whitney unpaired t-test. Results are shown as Mean ± Standard deviation (SD) unless otherwise stated. The difference in gender between the groups was assessed by a Chi-squared test. In the cleavage analysis of type III collagen, statistical significance between the different proteases was determined by ANOVA with multiple comparison testing using the Tukey test with type III collagen, without any protease present (COL3), as a reference. Difference in C3C levels between healthy controls and COPD patients was assessed using a two-tailed Mann-Whitney unpaired t-test. The correlation of C3C in serum and heparin plasma was assessed by linear regression analysis. Correlation between C3C level and age was performed using nonparametric spearman rank correlations. Differences were considered statistically significant if p<0.05. Asterisks indicate the following: *: p<0.05; **: p<0.01; ***: p<0.001; ****: p<0.0001. All statistical analyses were performed in GraphPad Prism v6 (Graph Pad Software, La Jolla, CA, USA) or MedCalc (Ostend, Belgium).

## Results

### Clone selection and characterization

**Fig 2:**
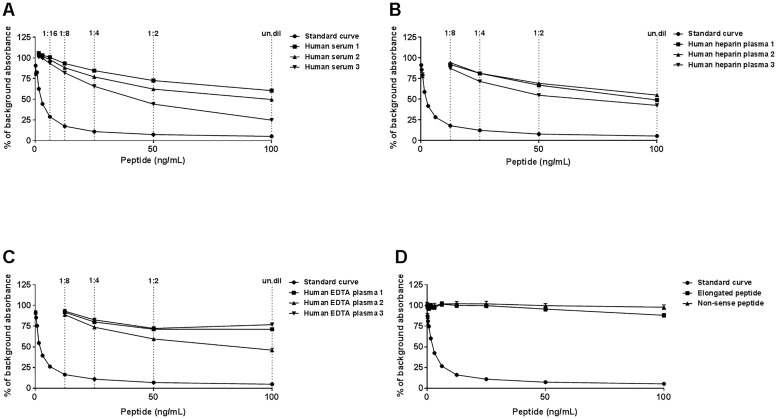
C3C ELISA runs showing typical standard curves and native reactivity against human serum, heparin and EDTA plasma. (A) Standard curve and inhibition of the competitive ELISA signal using healthy human serum, (B) human heparin plasma and (C) human EDTA plasma. The native material was run from undiluted and up to 8-fold diluted as indicated. (D) Neo-epitope specificity of the C3C ELISA was shown by comparing reactivity towards an elongated peptide, i.e. a peptide with an additional amino acid at the N-terminal generated by cleavage, a non-sense peptide, i.e. a peptide with a different sequence, and the standard peptide. The standard peptide (i.e. standard curve), elongated peptide, and non-sense peptide were diluted 2-fold from 100 ng/mL. The data is presented as percent (%) of background absorbance, which is the absorbance with only assay buffer present, as a function of peptide concentration.

To select the most optimal antibody producing hybridoma, supernatants were screened for reactivity against standard peptide and native material in an indirect ELISA. The peptide GLPGTGGPPG-K-Biotin was used to screen for reactivity. Based on reactivity, we selected antibody clone NB239-1D4, and determined it to be of the IgG1 subtype. Native reactivity was observed in human serum and plasma (Fig 2A–2C), and no inhibition was observed using the elongated peptide and non-sense peptide (Fig 2D).

### Technical evaluation

The measurement range was determined by calculating the LLOQ and ULOD, which provided a range of 0.731–68.33 ng/mL. Evaluation of the mean intra- and inter-assay variation yielded 6.3% and 10.66%, respectively (Table 3). Linearity of dilution was observed from undiluted to 1:4, undiluted to 1:4 and 1:2 to 1:4 for human serum, heparin plasma and EDTA plasma, respectively (Table 4). Spiking of standard peptide in human serum and high serum in low human serum resulted in a mean recovery of 161% and 85%, respectively (Table 5). Whereas neither high levels of hemoglobin nor lipids interfered with the measurements of the C3C analyte in human serum, the highest level of biotin (90 ng/ml) reduced the signal to 39% of levels without biotin present (Table 6). The stability of the analyte was acceptable during both freeze/thaw cycles (98%) and prolonged storage of human serum sample at 4 and 20°C (both 97%) (Table 7).

**Table 3:** Inter- and intra-assay variation for the C3C assay. As quality controls (QC1-5), human sera was used. The controls (CO1 and -2) included in every run of the C3C ELISA were also included. The variation was calculated as the mean variation between 10 individual runs of each sample run in double determination.

Sample	C3C (ng/ml)	Intra-assay variability %	Inter-assay variability %
**CO1**	1.28	2.98	12.79
**CO2**	24.8	9.83	15.85
**QC1**	0.85	9.56	12.15
**QC2**	1.37	8.73	9.21
**QC3**	2.04	4.39	8.38
**QC4**	7.29	3.88	6.28
**QC5**	20.1	4.59	10.00
**Mean**		6.28	10.67

**Table 4:** Percentage dilution recovery for the C3C assay using human serum and plasma samples. Samples were measured from undiluted to 1:4 diluted and linearity was assessed.

	Serum (n = 4)	Heparin plasma (n = 4)	EDTA plasma (n = 4)
**Undiluted**	100%	100%	-
**Dilution 1:2**	99	103	100%
**Dilution 1:4**	84	107	105
**Mean**	94	103	103

**Table 5:** Spiking recovery of standard peptide in human serum, and high serum in low serum. The recovery (RE%) was calculated as the percentage recovery of the measured amount in the sample alone. The experiment was performed for three separate healthy human control sera. The standard peptide was added in 2-fold dilutions starting from 50 ng/mL (StdB) and high serum was added in 2-fold dilution starting from 1:2.

Peptide in serum (n = 3)		High serum in low serum (n = 3)	
**Added Std**	**RE%**	**Added high serum**	**RE%**
**StdB**	-	**2x**	56
**StdC**	200	**4x**	81
**StdD**	184	**8x**	95
**StdE**	160	**16x**	93
**StdF**	142	**32x**	92
**StdG**	121	**64x**	94
**Mean**	161		85

**Table 6:** Interference of hemoglobin, lipids and biotin in human serum. The interfering substances were added in two concentrations (high and low) and compared to the sample without the interfering component. All data are shown as mean percent recovery (RE%) compared to serum alone.

	Hemoglobin		Lipids		Biotin	
	**mmol/L**	**RE%**	**mmol/L**	**RE**%	**ng/mL**	**RE**%
	0.000	100	0.00	100	0.0	100
	0.155	98	4.83	95	30.0	89
	0.310	105	10.98	93	90.0	39
**Mean**		102		96		76

**Table 7:** Analyte stability in human serum. The serum samples were either subjected to four freeze/thaw cycles or stored at 4 or 20°C for 0, 2, 4 and 24 hours. All data are shown as mean percent recovery (RE%) compared to baseline (ie, 1 freeze/thaw cycle and storage time 0 hours, respectively).

Freeze/thaw cycle	RE%	Storage time (Hours)	Stored at 4°C RE%	Stored at 20°C RE%
1	100	0	100	100
2	92	2	99	94
3	99	4	92	97
4	99	24	97	98
**Mean**	98		97	97

### C3C is specifically generated by cathepsins

**Fig 3:**
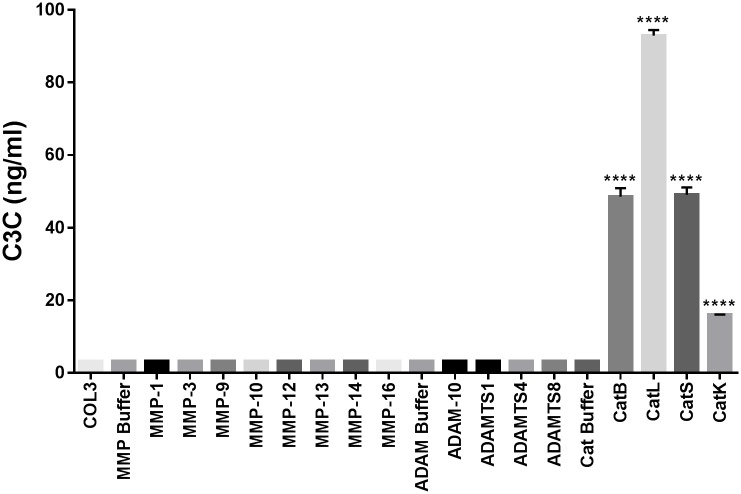
Generation of C3C by cleavage analysis of type III collagen. No reactivity was seen for either of the buffers or intact type III collagen (COL3). The only proteases generating C3C were the cathepsins B, L, S and K. Data are presented as mean ± SEM. Results below the detection limit were given the value of LLOQ. Statistical significance was determined by ANOVA with multiple comparison testing using the Tukey test with type III collagen, without any protease present (COL3), as a reference; ****p<0.0001.

To assess which proteases could generate the C3C neo-epitope a range of different proteases were incubated with type III collagen. Based on the cleavage analysis, the fragment of type III collagen is mainly generated by cathepsins B, L, S and, to a lesser extent, K (Fig 3). None of the other proteases were able to generate the C3C fragment at this condition.

### C3C levels are higher in COPD patients than in healthy controls

**Fig 4:**
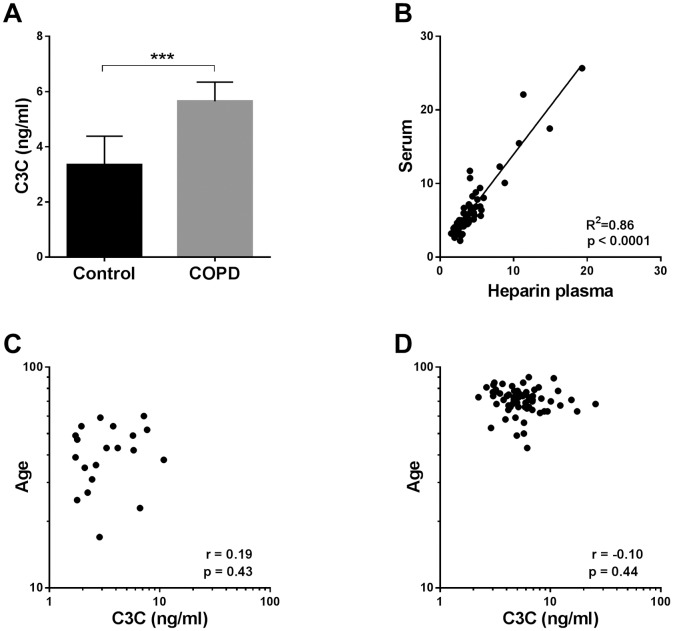
C3C levels in COPD patients and healthy controls. (A) Levels of C3C were measured in serum of patients with COPD (n = 68) and compared to levels in healthy controls (n = 11). Results are shown as geometric mean with 95% confidence intervals. Statistical significance between healthy controls and COPD patients was assessed using the two-tailed Mann-Whitney unpaired t-test. (B) The correlation of the C3C fragment levels in serum and heparin plasma was tested in the cohort of COPD patients (n = 68) by the use of linear regression analysis. Correlation between age and levels of C3C in the healthy controls (C) and COPD patients (D) was analyzed using nonparametric spearman rank analysis. *p<0.05, **p<0.01, ***p<0.001, ****p<0.0001.

As type III collagen is one of the most prominent collagens in the lung [1–3], and patients with COPD are believed to have higher cathepsin activity [6,7,13,14], samples from a previously described cohort of COPD patients were measured with the C3C assay and compared to levels in a group of healthy controls (Fig 4). The levels of C3C in were significantly higher in patients with COPD compared to healthy controls (Fig 4A, p = 0.0006). When comparing levels of C3C in serum and heparin plasma from the COPD patients, a highly significant positive correlation was observed (Fig 4B, R^2^ = 0.86, p<0.0001). Patients with COPD were significantly older than the healthy control group (Table 2, p<0.0001). To determine whether the difference between COPD patients and healthy controls was merely due to the difference in age, C3C levels were correlated to age in both the healthy controls (Fig 4C) and COPD patients (Fig 4D). No significant correlation between C3C levels and age was observed.

## Discussion

We developed and characterized the novel competitive ELISA C3C using a monoclonal antibody detecting neo-epitope fragments of type III collagen generated by the proteases cathepsins B, L, S and K. The C3C assay specifically recognized the human sequence GLPGTGGPPG, from residue 642 towards 651, and was able to measure fragments containing this sequence in human serum and plasma. The assay was robust and all technical parameters were accepted except when abnormally high levels of biotin were present. The biological relevance of this particular neo-epitope was confirmed in samples from a previously described cohort of COPD patients. Our data suggest that there is an elevated level of cathepsin B, L, S or K activity and/or an upregulated type III collagen production in patients with COPD giving rise to a higher level of type III collagen fragments containing the C3C neo-epitope.

As type III collagen is one of the most prominent proteins of the lung [1–3], fragments of this collagen in the circulation, such as C3C, could reflect a shift in the turnover of the lung tissue and could possibly be involved in airspace enlargement. The involvement of cysteine cathepsins in various diseases is a well-known phenomenon, and it is beyond the scope of this paper to discuss, but has been reviewed elsewhere [21]. In the lung, cathepsins are upregulated in patients with COPD [6,7], IPF [8] and lung cancer [9–11]. In patients with COPD it seems that both cathepsin S and L are involved in degradation of the extracellular matrix mediated by macrophages [13,14]. In transgenic mice overexpressing IL-13, matrix metalloprotease (MMP)- 2, -9, -12, -13, and -14, and cathepsins B, L, S, H, and K were upregulated and treatment with MMP and cysteine proteinase antagonists significantly decreased the emphysema [15]. This demonstrates that IL-13 causes emphysema through MMPs and cathepsins which might be part of the pathological alterations causing COPD. In another strain of transgenic mice, over-expression of IFN-γ caused cathepsin S-dependent inflammation, epithelial cell death, and airspace enlargement in the lung [16]. In the mice lung damage models utilizing bleomycin and silica, a high expression of cathepsin K was seen in macrophages and fibroblasts [22,23]. As overexpression of cathepsin K in transgenic mice led to a reduction of collagen deposition in the lung, Srivastava *et al* suggested that this cathepsin could have a role in the regulation of fibrosis [23]. Work performed by Buhling *et al*, showed that cathepsin K mRNA was upregulated in tumor versus normal tissues of the lung from the same patients, suggesting an involvement of this cathepsin in tumor invasiveness [11]. Based on the evidence listed above, we hypothesize that the C3C ELISA would be able to detect increased tissue remodeling observed in pulmonary disorders such as IPF, COPD and lung cancer. It could therefore be a useful tool in assessing the dynamic alterations taking place in the ECM of the lung mediated by cathepsin activity.

The assay could also be useful in other diseases where cathepsins B, L, S and K are known to play a role. In inflamed mucosa of patients with inflammatory bowel disease (IBD) higher levels of cathepsins B and L were observed in macrophages [24]. The C3C assay could therefore be used to assess the degree of cathepsin-mediated type III collagen turnover in patients with IBD.

Abundant evidence has been presented on the upregulation of cathepsins in cancer, and that the presence of increased cathepsin activity is correlated with malignant progression and poor prognosis of patients with breast, lung, colorectal cancer, and many other types of cancer [21,25–28]. In breast cancer, levels of cathepsins B and L were indicative of lymph node metastasis, poor prognosis, and poor survival [29–33]. In colorectal cancer, cathepsins B, L and S were involved in the pathological tissue turnover, and were associated with metastasis and prognosis [34–36]. In lung cancer patients, cathepsin B expression was shown to be a prognostic marker of shorter overall survival [37–40]. Combined, the increased activity of cathepsins B, L, S and K in the various types of cancer leads us to believe that the C3C assay would be a useful tool in assessing the cathepsin-mediated effects on the ECM turnover in cancer.

A limitation of the study was the significant difference in age between the COPD patients and the healthy controls (Table 2). Due to the large age difference it is not clear whether the observed difference in C3C levels is due to age or pathology. When correlating levels of C3C in healthy controls and COPD patients with age, there was no significant correlation in the isolated subgroups (Fig 4), suggesting that there is no correlation of C3C with age. Age-matched samples were not readily available, and the healthy controls were included to provide an indication on the serum levels of the investigated fragment in the normal population. Future studies with age- and gender-matched samples should be performed to assess the significance of the current findings.

Another limitation is the inhibition of the C3C ELISA by very high levels of biotin. The levels of biotin used to spike the samples in our interference tests are higher than expected in circulation of healthy individuals (0.034 to 0.089 ng/ml) [41]. Even in subjects taking long term biotin as a supplement, rises in biotin, ranging from 2.3–11.7 ng/ml [41], do not reach the levels tested for the assay which were 30 ng/ml (low) and 90 ng/ml (high). As there was no significant reduction in reactivity in the low sample (Table 6) we do not believe that biotin interference would pose an issue for the C3C ELISA in patient samples.

In conclusion, we have developed a robust and specific assay, which detects specific fragments of type III collagen, containing the neo-epitope generated by cathepsins B, L, S and K. Fragments containing the C3C neo-epitope were elevated in patients with COPD compared to healthy controls. The assay could potentially be used in other diseases with known, or suspected, increased cathepsin activity and high production of type III collagen, such as IBD and various types of cancer.

## Supporting Information

S1 Data Data for technical and biological validation of the C3C ELISA. All data used for technical and biological validation of the C3C ELISA can be found in the ‘S1 Data.xlsx’ file.
(XLSX)
